# Use of Instant Messaging Software in a German Hospital—An Exploratory Investigation among Physicians

**DOI:** 10.3390/ijerph191912618

**Published:** 2022-10-02

**Authors:** Sabine Sayegh-Jodehl, Rebecca Mukowski-Kickhöfel, Diane Linke, Claudia Müller-Birn, Matthias Rose

**Affiliations:** 1 Department of Psychosomatic Medicine, Campus Charité Mitte, Charité-Universitätsmedizin Berlin, 10117 Berlin, Germany; 2 Department of Psychosomatic Medicine, Campus Benjamin Franklin, Charité-Universitätsmedizin Berlin, 12203 Berlin, Germany; 3 Human-Centered Computing Research Group, Institute of Computer Science, Freie Universität Berlin, 14195 Berlin, Germany

**Keywords:** communication, text messages, instant messaging, healthcare, physicians, media competence, computer literacy, digital technology, Germany

## Abstract

Internationally, evidence exists that physicians use instant messaging services for communication tasks in everyday clinical practice However, there are only few data on physicians in Germany in this regard. Therefore, at the initiation of our project “DocTalk-Dialog meets Chatbot: Collaborative Learning and Teaching in the Process of Work”, we conducted a stakeholder survey with an exploratory research approach. The aim was to gain initial insights into use of instant messaging software and attitudes towards data security and advantages and disadvantages before implementing a data-secure in-house messaging platform. *N* = 70 physicians at Charité-Universitätsmedizin Berlin completed an exploratory questionnaire with closed and open-ended questions. Quantitative data were analyzed using descriptive statistics and qualitative data using thematic analysis. The use of messenger software was not widespread in the sample studied. Physicians most frequently used face-to-face contact for communication. On average, up to ten instant messages were exchanged per day, mainly among colleagues, to answer mutual questions, and to send pictures. With a high awareness of privacy-related restrictions among participating physicians, advantages such as fast and uncomplicated communication were also highlighted. An instant messenger solution that complies with the German data protection guidelines is needed and should be investigated in more detail.

## 1. Introduction

For communication tasks in a digitized hospital, the literature states a positive benefit for messenger software, especially for collegial communication between employees, interdisciplinary consultation, and continuing medical education [1,2]. There is evidence in the international scientific literature that physicians use instant messaging services as a tool for clinical case discussions, interactions between healthcare providers and patients, and knowledge dissemination [3]. However, there are few studies examining the utility of text-based communication software among physicians in everyday clinical practice in Germany. In 2018, the German Data Protection Institute identified through a survey that 98 percent of the 353 participating physicians use WhatsApp^®^, Facebook Messenger, and other communication apps. More than half of those surveyed use these applications, among other things, to send patient data, such as laboratory findings or even X-ray images, to colleagues. When asked about data protection precautions, 84 percent of the doctors in question answered that they hide the identity of the patients [4]. 

From the perspective of German data protection and data security, the use of private messengers was classified as unsuitable for use in hospitals [5,6]. The Data Protection Conference (DSK) published a white paper in November 2019. This summarized technical data protection requirements for messenger services in hospitals and regulated the necessary framework conditions for their use in Germany [7]. To date, no instant messenger is available in Germany that would meet these requirements.

In order to become better acquainted with the potential of digital communication for medical education, training, and continuing education even before the official launch of a data safe messaging service throughout Germany’s healthcare sector, the project “DocTalk-Dialog meets Chatbot: Collaborative Learning and Teaching in the Work Process” has set itself the goal of testing digital communication and learning paths in a German hospital. The project also intends to enable physicians to use digital communication tools for professional purposes in a reflective manner against the background of the German data protection law and professional ethics [8]. Over a period of three years, “DocTalk” is pursuing a holistic teaching–learning concept with an interdisciplinary network of medicine (Department of Psychosomatic Medicine Charité-Universitätsmedizin Berlin, headed by Prof. Dr. med. Matthias Rose), media didactics (Fernuniversität Hagen, headed by Prof. Dr. phil. Sandra Hofhues), and human–computer interactions (Institute of Computer Science, Human-Centered Computing Research Group, Freie Universität Berlin, headed by Prof. Dr. Claudia Müller-Birn). The project is funded by the German Federal Ministry of Education and Research (BMBF) under the funding code 01PG20002 [8].

Since the project could not rely on a German-approved messaging platform for data-secure operation in the hospital, it was necessary to implement a secure messenger platform as part of the project. With a view to low cost and an open-source policy of the DocTalk project, we chose Mattermost^®^ as instant messaging service [9]. The software is installed and operated on a virtual server within the clinical IT infrastructure of the Charité (on-premises) to be able to establish a maximum of data security. Mattermost^®^ allows the chatting with individuals as well as group chats organized in “channels”. Mattermost thus functions similarly to the market-leading proprietary software Microsoft Teams^®^ and Slack^®^ [10,11]. This instant messaging software has been made available to physicians from the four departments involved in the project (emergency medicine, gastroenterology, general surgery and psychosomatics) at the Charité University Hospital for the duration of the project.

Using an exploratory research approach, we conducted a stakeholder survey at the beginning of the DocTalk project, prior to the implementation of a project-specific instant messaging platform. The goal was to gain initial insight into the status quo of communication software use in the clinical setting among participating physicians and to obtain indications of attitudes toward safety and use, as well as into key perceived advantages and disadvantages. This article reports the results of that survey. A final survey using the same scales is planned for the end of the project period after using the implemented data secure messaging platform.

## 2. Materials and Methods

### 2.1. Participants and Procedure

We asked physicians from the four departments of Charité-Universitätsmedizin Berlin involved in the DocTalk project by e-mail and via mailing list to participate in the planned survey (about 145 physicians). Participation was voluntary and pseudonymous. *N* = 138 clinicians started the online survey. For the analysis, we only used only fully completed and consented records. This resulted in *N* = 70 data sets. The mean age was 36.63 years (SD = 7.98), and 57.14% were female. *N* = 53 were residents, and *N* = 17 were senior physicians.

At the beginning of the online survey, a brief description of the questionnaire, details about pseudonymity, privacy, and intent to publish were provided. Informed consent was obtained with a confirmation button on the second page. Study approval from the Ethics Committee of the Charité Universitätsmedizin Berlin is available under number EA4/031/21.

Between May and June 2021, study data were collected and managed using REDCap electronic data capture tools hosted at Charité Universitätsmedizin Berlin [12,13]. REDCap (Research Electronic Data Capture) is a secure, web-based software platform designed to support data capture for research studies.

### 2.2. Exploratory Questionnaire

First, we collected general information on gender, age, and position (resident or senior physician). Second, we asked physicians how they primarily communicate with their colleagues in the daily clinical routine. We investigated the extent of use of instant messengers in the clinical setting, attitudes toward safety, attitudes toward their use, main advantages, and possible disadvantages using an adapted version of the questionnaire by Nikolic et al. [14] translated into German. Example questions and answer options are shown in Table 1.

Finally, we asked the participants about their personal support needs with regard to their own digital media literacy. The following constructs were also surveyed in the present survey: the conditions of continuing education at the Charité-Universitätsmedizin Berlin, media pedagogical competence, learning and teaching conditions in continuing medical education, and perceived medical competence.

These constructs primarily serve the (media) pedagogical perspective of the project. We decided to focus on the use of instant messenger apps in this publication to counter the lack of previous German studies on this topic. The overview of the items (19 questions in total, 11 closed questions, and 8 open questions) can be found at the end of this article as Appendix A.

### 2.3. Data Analysis

A quantitative descriptive analysis of responses to closed-ended questions was performed in R Studio [15] and figure s were generated using the ggplot2 package [16]. For the qualitative analysis of the free-text responses data, the 70 final datasets were transferred to MAXQDA [17] for computer-assisted qualitative thematic analysis. The qualitative data were left in German for analysis and only subsequently translated into English for publication. The quality of our work was supported by the use of Silver and Lewins’ guide for computer-assisted qualitative data analysis [18]. In a first step, the free-text answers were read and interpreted by the first author (S.S.-J.), after which a test code system was created using inductive categorization and manually assigned to the free-text answers. As a second step, the test code system was reviewed by a second researcher from the team and checked for consistency. Next, a consensus code system was created through discussion between the researchers. Code frequencies were determined using MAXQDA. The figures were created using Microsoft Excel [19]. The number of free-text responses received per open-ended question can be found in Table 2. Most answers were very short, single words or lists of keywords, so that several codes could often be assigned to single answers, nevertheless. Finally, the results of the quantitative and qualitative analysis were interpreted descriptively. Due to the small sample size, no generalization or inferential methods were used.

**Figure 1:**
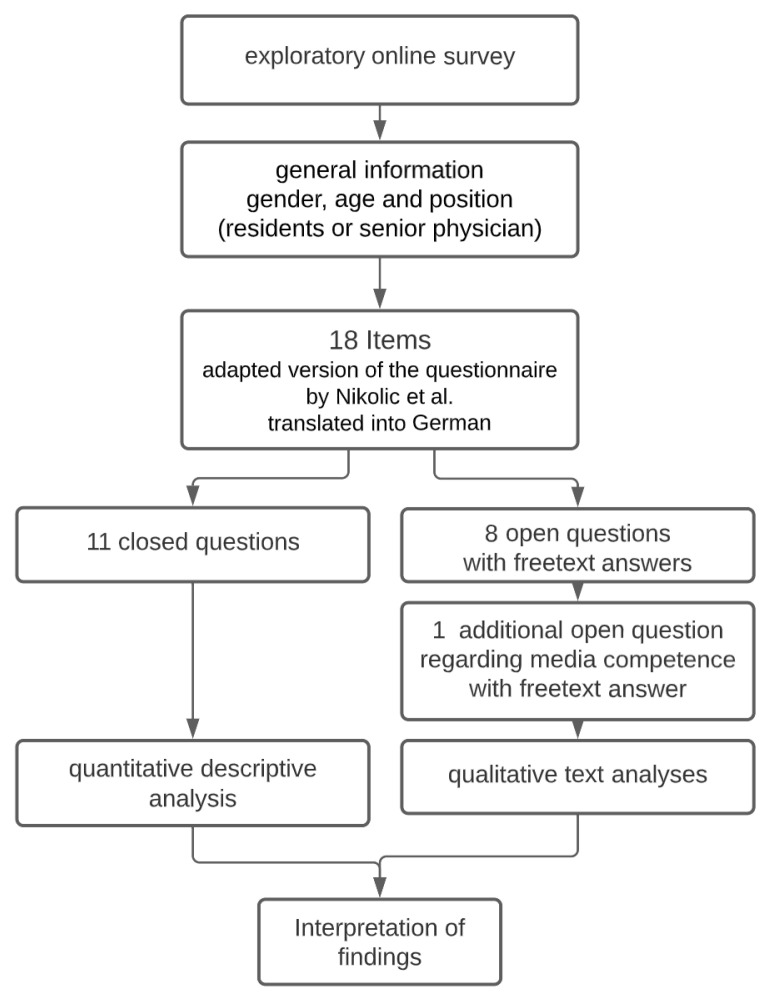
Flowchart of methodology (adapted version of the questionnaire by Nikolic et al. [18]).

The flow of the methodology can be seen in a flow chart in Figure 1.

## 3. Results

Due to the low number of completed data sets (*N* = 70), we opted for a descriptive presentation of the results.

### 3.1. Usage of Instant Messaging Software

No one primarily used communication apps in clinical practice. Face-to-face conversations (51.43%) were the most important type of communication. In second place, physicians used phone calls (41.43%). In the third position, e-mail was indicated as the main path of communication (7.14%). When communication apps were used, there was a difference between private use and use for official matters. Although all physicians reported using communication apps for private purposes, *N* = 9 physicians reported not using a communication app in clinical practice. Most physicians use WhatsApp^®^ for private communication and Microsoft Teams^®^ (In June 2019, Microsoft Teams was introduced to the departments of the Charité Hospital for general work-related communication, with the exception of the transmission of patient information (corresponds to data with high protection requirements)) for their work-related communication. Some participants admitted to having used private messengers in clinical settings before. On average, physicians in clinical practice send and receive zero to ten messages per day via communication apps.

### 3.2. Specific Use of Instant Messaging Software in Clinical Practice

#### 3.2.1. Purpose of Use

**Figure 2:**
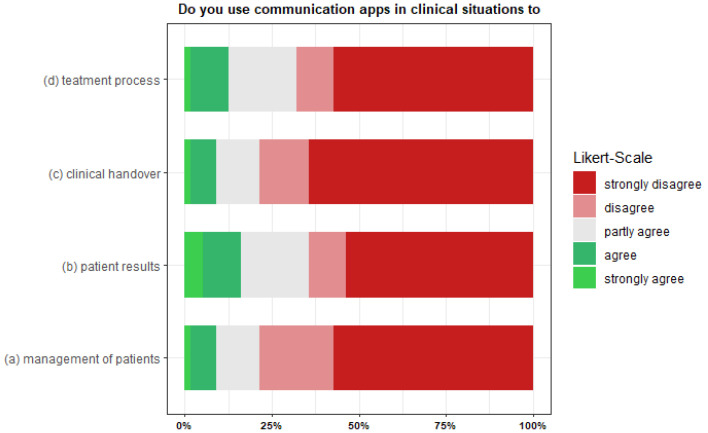
“Do you use communication apps in clinical situations to (**a**) communicate with colleagues (**b**) update colleagues about patient results? (**c**) facilitate clinical handover? (**d**) inform colleagues about the treatment process?” (*N* = 70).

When asked for what purpose physicians use instant messaging software, more than 50% disagreed (strongly) with communicating with colleagues about patient management (62.86%), patient outcomes (51.43%), clinical handoffs (62.86%), or the treatment process (54.29%) via a communication app. In addition, 10–15.71% partially agreed to use it for these purposes. The minority of physicians (strongly) agreed with the statements of using communication apps to communicate about patient management (7.14%), patient outcomes (12.86%), clinical handover (7.14%), or the treatment process (10%). Figure 2 visualizes agreement or disagreement on the used Likert scale for the listed purposes: (a) to coordinate treatment planning with colleagues, (b) to inform colleagues about examination results, (c) to make a handover to their colleagues, and (d) to inform their colleagues about the progress of treatment.

We also asked the physicians in a free text format what they use the communication apps for (Q7, “What do you use communication apps for in your daily clinical routine?”, *N* = 45/70). Most frequently, they used communication apps for organizational arrangements, such as scheduling shifts, or for organizing students’ classes and breaks. In addition, physicians used them for videoconferencing, especially for team and research meetings, teaching sessions, and lectures for continuing education.

#### 3.2.2. Interlocutors

**Figure 3:**
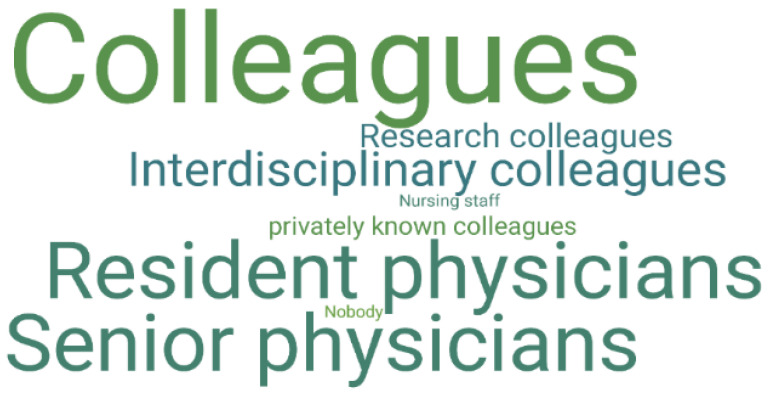
This figure shows a code cloud for the subgroups of residents (*N* = 53/70) to the question “With whom do you communicate via communication apps in everyday clinical practice?” (Q8). The size of the words linearly reflects the frequency of codes assigned to the responses in the subgroup. Most of the responses contain more than one group of people.

**Figure 4:**
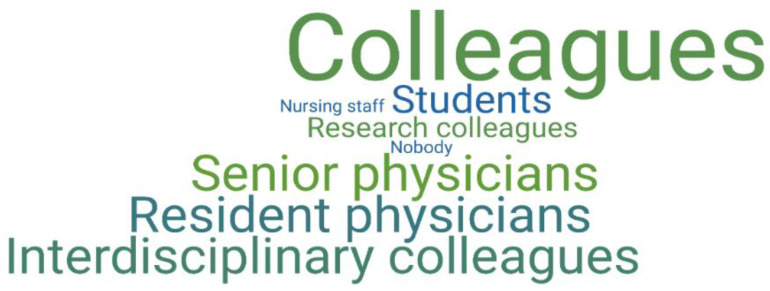
This figure shows a code cloud for the subset of senior physicians (*N* = 17/70) to the question “With whom do you communicate via communication apps in everyday clinical practice?” (Q8). The size of the words linearly reflects the frequency of codes assigned to the responses in the subgroup. Most of the responses contain more than one group of people.

“Colleagues” in particular were mentioned as interlocutors via instant messaging software (23 times in 42 given responses) (Q8, “With whom do you communicate via communication apps in the daily clinic routine?” *N* = 42/70). When considering the two subgroups of residents and senior physicians, residents reported being more likely to communicate with senior physicians and senior physicians reported communicating with both residents and colleagues in other specialties involved in patient care. Figure 3 shows a code cloud for the subset of residents and Figure 4 displays a code clouds for the subset of senior physicians.

#### 3.2.3. Sent Information

**Figure 5:**
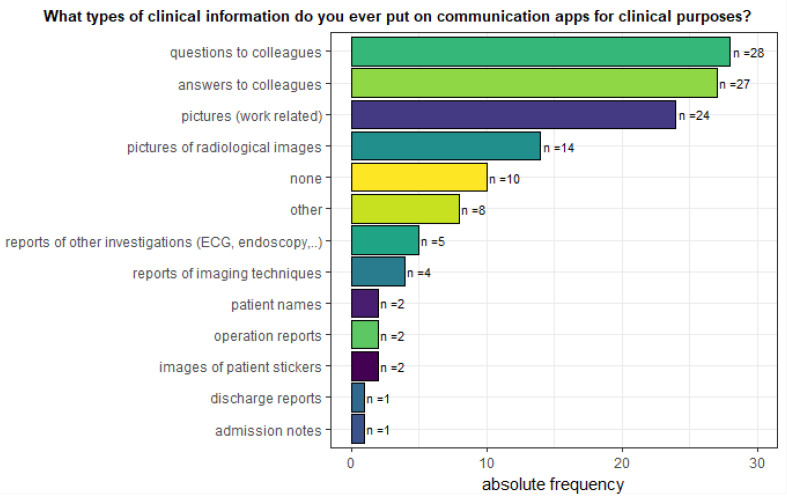
The figure shows the answers to the following multiple-choice question: “What types of clinical information do you ever put on communication apps for clinical purposes? (You can choose more than 1 response)”.

In addition, we requested that physicians indicate what clinical information they had ever sent via a communication app (Figure 5). As shown in Figure 5, the main purpose of communication via instant messaging software was to answer mutual questions between colleagues. Second, physicians used the communication app for work-related pictures (*N* = 24). Only *N* = 2 physicians ever sent patient names, surgical reports, or pictures of patient stickers via a communication app. Another *N* = 10 participants never sent any of the possible responses via communication app.

#### 3.2.4. Group Chat Usage

Of the physicians, 55.71% reported having participated in one or more work-related group chat on communication software, while 22.86% of participants did not answer the question. Of the physicians who reported having participated in work-related group chats, the average was in two to three groups (M = 2.51, SD = 1.5).

#### 3.2.5. Perceived Advantages and Barriers of Instant Messaging Software in Clinical Practice

The majority of physicians named “fast” (27 times in 50 given answers) and “uncomplicated / simple” transmission of messages (12 times in 50 given answers) as advantages of instant messaging software to the open question “What do you see as the benefits of using communication apps in your daily clinical practice?” (Q16, *N* = 50/70). The inductively assigned codes could for the most part be kept identical to the answered keywords. The answers mostly contained lists of keywords. The answers could be divided into three groups of codes: Functionality, Function, No benefits. The absolute frequencies of the codes assigned can be seen in Table 3.

Most participants cited “data protection” (36 times in 51 given answers) and “data security” (7 times in 51 given answers) as current barriers to the use of instant messaging software in everyday medical practice. In addition, physicians mentioned “lack of acceptance and low usage” as barriers (6 times in 51 given answers). They also feared a “loss of information” and “less personal contact” with colleagues in response to the open-ended question “What challenges do you see in using communication apps in everyday clinical practice?” (Q17, *N* = 51/70).

#### 3.2.6. Usage Evaluation

**Figure 6:**
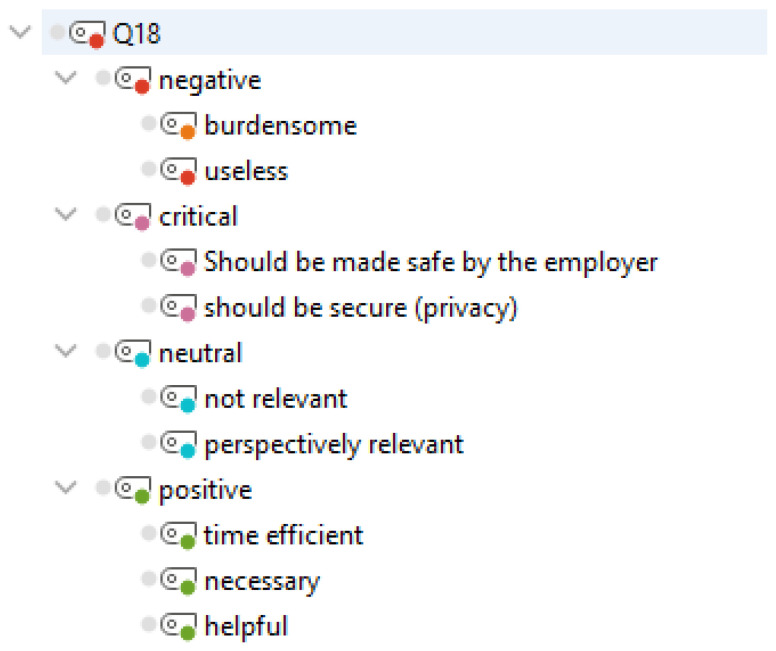
System of assigned codes and sub codes to the question “How do you evaluate the use of communication apps in everyday hospital life?” (Q18, *N* = 49/70).

As a summary assessment, we asked the physicians in an open question for their opinion how they evaluate the use of communication apps in the daily hospital routine. (“How do you evaluate the use of communication apps in everyday hospital life?”, Q18, *N* = 49/70). The inductive developed code system and grouping into categories is shown in Figure 6.

*N* = 23 physicians rated the use of instant messaging software as “positive”, *N* = 18 as “critical”, *N* = 15 as “neutral”, and *N* = 4 as “negative”.

Example segments with assigned codes to assess the use of communication apps in everyday hospital life are shown in Table 4.

### 3.3. Self-Reported Need for Media Competence Development

To determine the need for further training on the topic of media competence, we asked the physicians to self-assess with the open-ended question: “What support needs do you see with regard to your own media competence?” (Q19, *N* = 34/70). *N* = 13 physicians indicated no need for support, while *N* = 12 expressed a need to develop their media literacy, particularly in the area of data protection and IT security, and a desire for usage guidelines.

## 4. Discussion

### 4.1. General Discussion

Seventy physicians were surveyed about the use of instant messaging software in their daily medical work. The descriptive analysis showed that the most frequent communication channel among the surveyed hospital physicians is face to face contact. On average, physicians reported sending and receiving up to ten messages per day via text-based communication software. In contrast to the study by Nikolic et al. [14], there was no predominant use of messenger services in the present sample at the time of data collection. On the one hand, the physicians in the sample showed an increased awareness of privacy-related difficulties when using messenger apps for private purposes, such as WhatsApp^®^. On the other hand, the clear advantages of messenger services with the possibility of fast and uncomplicated communication as well as the possibility of sending pictures were mentioned.

Although there is currently no data-protected messenger service for medical purposes in Germany, the participating physicians reported the use of instant messaging software. Here, it can be assumed that it is mainly communication that does not contain patient data, as Microsoft Teams^®^ was introduced at Charité-Universitätsmedizin Berlin in June 2019 for work-related communication without sensitive data, such as patient information. A central procedural instruction is available in this regard. Given the increasing specialization and a shortage of specialists in the medical landscape, effective and fast communication including data transfer will become indispensable in the future [20]. Currently, the minority of physicians in the study agree with statements about using instant messaging software to communicate about patient management, patient findings, clinical handoffs, or the treatment process. In the present sample, colleagues were mentioned as the most frequent interlocutors, but here it is not clear who is behind the designation “colleagues”; these could also be employees of other occupational groups. Further research should be extended to other professional groups and other areas of the medical system. Due to the privacy risks associated with sending messages via instant messenger software, it can be assumed that there is underreporting in this study. In further studies, a significantly larger sampling could make it easier for respondents to provide more precise information. The DocTalk project and the study described here were planned before the pandemic and started at the beginning of the pandemic in Germany, so that the survey in June 2021 could already no longer generate a pandemic-independent picture because, as described by Schütze et al. [21], the occurrence of the pandemic required additional necessary communication and thus could have had an influence on physicians’ opinions.

Krefting describes a great need for further training for healthcare professionals in Germany [22]. Given the high level of awareness of data protection obstacles among the study participants, it can be assumed that the need for further training in this area is low, so the need for training on data protection was also mentioned by only a minority (Q19). Other areas of media literacy described in Ebert’s [23] article, such as the ability to operate devices, evaluate media, select and critically reflect on information, and change media when necessary, were not mentioned by the physicians surveyed. This suggests that physicians either do not have such a comprehensive understanding of media literacy or that existing training and support needs, e.g., for familiarization with newly introduced technologies, are not anticipated at all. According to Bauer et al. [24], the use of buzzwords, such as media literacy, runs the risk of omitting the necessary situation- and target group-specific explanation of the terms in the context of medicine considered here.

### 4.2. Strengths and Limitations

Given the small sample size and the lack of previous studies on the use of instant messaging software in healthcare in Germany, we emphasize the need for further research in this area. Therefore, we see a major limitation of this study in the small sample size, which led us to limit ourselves to a descriptive presentation of the results instead of a causal analysis. Due to the project schedule, we had to decide against a broader data collection on the status quo of instant messenger usage. Furthermore, the present study is limited to the sample of physicians and the hospital setting. A study of other professionals who, for example, use more imaging modalities in their routine clinical practice and the inclusion of other health care settings outside the hospital could yield complementary results. Despite all its limitations, the strength of the present study lies in the attempt to approach the topic scientifically at all, even if this is quite delicate due to data protection considerations in Germany.

### 4.3. Further Implications

In Germany, the overall responsibility for the central platform for digital applications in the German healthcare system lies with gematik GmbH (National Agency for Digital Medicine) [25]. The gematik GmbH has officially announced that it has selected the software library Matrix an open standard on which it is data-secure and fully interoperable instant messaging standard—the TI Messenger—will be based [26]. With TI Messenger, gematik is building a nationwide decentralized private communications network that can potentially support more than 150,000 healthcare institutions in Germany [27]. As Kumar et al. [28] describe, healthcare data is particularly sensitive because its disclosure could lead to the exposure of the patient’s identity and medical conditions. Therefore, we agree with the authors that effective security mechanisms are needed for healthcare organizations. From the end of 2023, TI Messenger will provide end-to-end encrypted VoIP/video and messaging services for the entire healthcare sector, enabling the exchange of healthcare-related data, images, and files [27]. In this context, however, the question arises as to how clinically relevant information exchanged in chat histories should be handled with regard to an archiving obligation of patient records [29]. The gematik TI Messenger is planned together with an address book of all health professionals in Germany, so that the participating physicians can be reached asynchronously for exactly this purpose. Since this sample primarily sought conversation and collegial advice through the messaging software, it can be assumed that the introduction of a functional and data-secure TI Messenger will meet with great approval among healthcare actuators in Germany. As shown in our study, it would be particularly valuable to aim for a nationwide survey before the introduction of the TI-messenger in order to be able to use as baseline measurement for comparisons after introduction. In the more distant future, the TI Messenger will probably also offer function for communicating with patients. In other countries, such as China, SMS already was tested during the COVID-19 pandemic as an information channel on health-related topics for the population [30]. This option could also be implemented in Germany via TI messaging but would require further research and place additional demands on the media skills of all involved. Krefting [22] already welcomes the development of the TI Messenger to minimize the use of messenger software as workarounds for otherwise cumbersome processes, thus taking the real needs of healthcare workers seriously and aiming for a legally compliant solution. In addition to recording the current level of use, the need for media competence training should be examined in a more differentiated manner and expanded to include computer-mediated communication, as this will become important for digital communication in interdisciplinary teams and doctor–patient communication. This is because patients will also increasingly develop a desire for digital communication with their physicians.

## 5. Conclusions

In view of the question of how instant messenger services are used in a German hospital, the study takes up a difficult topic in terms of applicable data protection regulations in Germany. An exploratory survey showed that physicians in the sample studied currently make very little use of instant messenger solutions in their clinical work. Face-to-face contact is still the most common form of communication, followed by telephone contact. Instant messengers were used in contact with colleagues to make organizational arrangements, such as scheduling shifts, organizing student classes, and attending research conferences via video telephony. Treatment progress, handoffs, or treatment outcomes were shared rarely via instant messenger. It became clear that the participating physicians were aware of privacy regulations but still saw advantages of instant messaging in its quick and uncomplicated use. A possible outlook in Germany seems to be the introduction of a uniform data-secure communication standard for the healthcare sector and the introduction of the TI messenger. However, as the study presented here shows, there is still a way to go from a rather personally colored use with known colleagues to a meaningful, professionally used communication solution, e.g., for interdisciplinary teams and beyond the boundaries of the hospital. With few studies currently available, more research is needed in this area to make the use of digital communication tools truly useful for the digitalized medicine of tomorrow and to determine the need for institutional support and the training needs of the professional groups involved.

## Figures and Tables

**Table 1:** Example questions and answer options.

Item	Possible Answers
How do you mainly communicate with your colleagues in your daily clinical routine?	Telephone|E-mail|Communication apps|Direct personal contact|SMS|Other
Do you use one or more of the following communication apps privately|in your daily clinical routine?	WhatsApp|Signal|Telegram|Snapchat|Discord|Facebook Messenger|Skype|Slack|Viber|Microsoft Teams|Threema|Ginglo (SIMSme)|Wire|WeChat|Line|Siilo|Zoom|Mattermost|Matrix|Other|I don’t use any communication apps
Please indicate how many messages you send|receive on average via communication apps in your daily clinic routine.	none|0–10|11–20|21–30|31–40|41–50| > 50
Do you use communication apps in clinical situations to (a) communicate with colleagues about the management of patients (b) update colleagues about patient results? (c) facilitate clinical handover? (d) inform colleagues about the treatment process?	5-Point scale from “strongly disagree” to “strongly agree”
What do you see as the benefits|challenges of using communication apps in your daily clinical practice?	free text answer

**Table 2:** Number of free-text responses received per open-ended question.

No.	Question	Possible Answer	*N* = x/70
Q7.	What do you use communication apps for in your daily clinical routine?	Free text answer	45
Q8.	With whom do you communicate via communication apps in the daily clinic routine?	Free text answer	42
Q9.	In which situations do you use communication apps in your daily clinical routine?	Free text answer	33
Q15.	I have sent the following other clinical information via a communication app	Free text answer	7
Q16.	What do you see as the benefits of using communication apps in your daily clinical practice?	Free text answer	50
Q17.	What challenges do you see in using communication apps in everyday clinical practice?	Free text answer	51
Q18.	How do you evaluate the use of communication apps in everyday clinical practice?	Free text answer	49
Q19.	What support requirements do you see for yourself personally with regard to your own digital media competence?	Free text answer	34

**Table 3:** Absolute frequencies of the codes assigned (Q16) “What do you see as the benefits of using communication apps in your daily clinical practice?” (*N* = 50/70).

Functionality		Functions		No Benefits	
Code	x/50	Code	x/50	Code	x/50
fast	27	exchange with colleagues	8	unnecessary/none	10
uncomplicated/simple	12	accessibility of a group	7	distracting	3
asynchronous response option	9	Video telephony	7		
with high availability	7	sending pictures	6		
Bridging of spatial distance	4	Share screen	5		
effective	4	memory for information	4		
reliable	3	Sending text	3		
less disturbing	3	Transmission of findings	3		
		interdisciplinary exchange	2		
		accessibility of the background service	2		

**Table 4:** Example segments with assigned codes (Q18) “How do you evaluate the use of communication apps in everyday hospital life?”.

Document Number	Segment	Code/Sub Code
131	*“Good should be made more”*	**positive**
44	*“Practical and labor saving when used professionally”*	**positive**/helpful
oa_132	*“Helpful and time efficient”*	**positive**/time efficient
oa_37	*“Meanwhile indispensable”*	**positive**/necessary
27	*“not very relevant at the moment”*	**neutral**
86	*“neutral”*	**neutral**
31	*“Great potential, but currently critical due to lack of software adapted to legal framework conditions and lack of implementation by management level --> Frequently either (data protection) legally critical use or corresponding restraint and non-use.”*	**neutral**/not yet relevant **critical**/should make employer safe **critical**/should be safe (data protection)
119	*“If there was a secure app established at Charité, I would like to use the path more and see great potential here.”*	**neutral**/perspective relevant **critical**/should be safe (data protection)
oa_75	*“Critical, it is essential that patient rights and data protection are preserved.”*	**critical**/should be safe (data protection),
99	*“additional channel that needs to be “checked””*	**negative**
	*“bad”*	**negative**

## Data Availability

The data presented in this study are available on request from the corresponding author.

## Appendix A

**Table A1:** Translated questionnaire of the study.

No.	Question	Possible Answer
1	How do you mainly communicate with your colleagues in your daily clinical routine?	Telephone|E-mail|Communication apps|Direct personal contact|SMS|Other
2	Do you use one or more of the following communication apps privately?	WhatsApp|Signal|Telegram|Snapchat|Discord|Facebook Messenger|Skype|Slack|Viber|Microsoft Teams|Threema|Ginglo (SIMSme)|Wire|WeChat|Line|Siilo|Zoom|Mattermost|Matrix|Other|I don’t use any communication apps
3	Do you use one or more of the following communication apps in your daily clinical routine?	WhatsApp|Signal|Telegram|Snapchat|Discord|Facebook Messenger|Skype|Slack|Viber|Microsoft Teams|Threema|Ginglo (SIMSme)|Wire|WeChat|Line|Siilo|Zoom|Mattermost|Matrix|Other|I don’t use any communication apps
4	Please indicate how many messages you send on average via communication apps in your daily clinical routine.	none|0–10|11–20|21–30|31–40|41–50| > 50
5	Please indicate how many messages you receive on average via communication apps in your daily clinical routine.	none|0–10|11–20|21–30|31–40|41–50| > 50
6	Do you use communication apps in clinical situations to (a) communicate with colleagues about the management of patients (b) update colleagues about patient results? (c) facilitate clinical handover? (d) inform colleagues about the treatment process?	5-Point scale from “strongly disagree” to “strongly agree”
7	What do you use communication apps for in your daily clinical routine?	Free text answer
8	With whom do you communicate via communication apps in the daily clinic routine?	Free text answer
9	In which situations do you use communication apps in your daily clinical routine?	Free text answer
10	Are you in one or more communication apps in a “group” that primarily consists of a work team? (e.g., “ENT doctors ward...”; “Stroke Unit Team”;...)	yes/no
11	In how many clinic-related “groups” are you in the communication app?	Number
12	I communicate regularly in clinic-related	small groups (3–5) medium groups (5–12) large groups (>12)
13	I communicate via communication apps with individuals. in a group.	very rarely|rarely|occasionally|often|very often
14	What types of clinical information do you ever put on communication apps for clinical purposes? (You can choose more than 1 response)	Images of patient stickers Patient names Patient insurance numbers Pictures (work related) Admission notes Discharge letters Imaging reports Pictures of radiological images Reports of other investigations (echocardiography; endoscopy) EEGs Pathology reports Microbiology reports Questions to colleagues Operation reports Answers to colleagues Other None
15	I have sent the following other clinical information via a communication app	Free text answer
16	What do you see as the benefits of using communication apps in your daily clinical practice?	Free text answer
17	What challenges do you see in using communication apps in everyday clinical practice?	Free text answer
18	How do you evaluate the use of communication apps in everyday clinical practice?	Free text answer
19	What support requirements do you see for yourself personally with regard to your own digital media competence?	Free text answer
